# Sensor Fault Detection and Signal Restoration in Intelligent Vehicles

**DOI:** 10.3390/s19153306

**Published:** 2019-07-27

**Authors:** Yeun-Sub Byun, Baek-Hyun Kim, Rag-Gyo Jeong

**Affiliations:** Korea Railroad Research Institute, 176, Cheoldobangmulgwan-ro, Uiwang, Gyeonggi-do 16105, Korea

**Keywords:** fault detection, sensor fault, signal restoration, intelligent vehicle, autonomous vehicle, kinematic model

## Abstract

This paper presents fault diagnosis logic and signal restoration algorithms for vehicle motion sensors. Because various sensors are equipped to realize automatic operation of the vehicle, defects in these sensors lead to severe safety issues. Therefore, an effective and reliable fault detection and recovery system should be developed. The primary idea of the proposed fault detection system is the conversion of measured wheel speeds into vehicle central axis information and the selection of a reference central axis speed based on this information. Thus, the obtained results are employed to estimate the speed for all wheel sides, which are compared with measured values to identify fault and recover the fault signal. For fault diagnosis logic, a conditional expression is derived with only two variables to distinguish between normal and fault; further, an analytical redundancy structure and a simple diagnostic logic structure are presented. Finally, an off-line test is conducted using test vehicle information to validate the proposed method; it demonstrates that the proposed fault detection and signal restoration algorithm can satisfy the control performance required for each sensor failure.

## 1. Introduction

The desire for convenient and safe passenger transportation has increased the need for automated and intelligent automobiles. Consequently, the function of autonomous navigation has been introduced for passenger safety and convenience, and various in-vehicle devices such as ultrasonic sensors, radars, cameras, and actuators have been installed to detect the surrounding environment, recognize information, and control the motions of the vehicle. In addition, speed sensors, steering angle sensors, gyroscopes, and acceleration sensors can be installed to measure the vehicle operation state and movement and to use the gathered information for control. If faults occur in these sensors or actuators during automatic running, the vehicle may deflect from its route or fail to conduct the precise operation required for control, which may lead to an accident. To prevent accidents caused by these faults, technologies applying the soft computing method in fault detection (FDI) and fault tolerant control (FTC) of vehicles are garnering attention in academia and industry. In conjunction with these developments, various ideas and techniques for FDI/FTC methods, including neural network and fuzzy approaches, are presented [1,2,3]. To this end, the main purpose is to prevent or mitigate deterioration of the control performance of the system caused by a failure. In the field of fault diagnosis, various studies have been conducted to compensate or detect the fault sensor information by combining it with information of various sensors [4,5,6]. In a system in which a vehicle is driven by an electric motor, a frequency domain analysis technique may be applied in the fault diagnosis of the electric motor, whereas a frequency component analysis usually deals with the diagnosis of physical faults in rotating machinery [7].

Although studies on fault detection are particularly important for the aircraft sector [8,9,10,11,12,13], as even minor aircraft faults can lead to serious accidents, the increasing interest in autonomous vehicles has inspired numerous investigations of fault detection in the automotive sector [14,15,16,17,18,19,20,21,22,23]. Na et al. [24] applied residual sensitivity as a threshold for predicting the occurrence of vehicle sensor failure, while Emirler et al. [25] employed a virtual sensor featuring a velocity-scheduled Kalman filter to characterize vehicle kinematics and estimate the yaw rate. Huang and Su [26] devised a model-based fault detection and isolation scheme considering disturbance and noise to diagnose single sensor faults in intelligent navigation systems, while analytical redundancy and nonlinear transformation were also used to generate residual values used to detect embedded sensors in intelligent vehicles [27].

In these fault-related studies, when a fault is detected, the fault signal has been largely recovered using either direct or analytical redundancy [28]. In practical environments, analytical redundancy methods are studied and used because of the low cost or installation space required. This analytical fault detection method can be divided into a data-based method and signal model, a model-based method, and knowledge-based method [13]. The data-based method and signal model are applied to compare and analyze the characteristics of data [29]. The model-based method is based on the mathematical model of the target system [30]. Knowledge-based methods are implemented using expert systems or fuzzy logic [31]. Among these methods, the model-based method that is acknowledged in this paper is classified into parity equations [32,33], parameter estimation methods [34], and observer-based methods [35,36]. In addition, this study focuses on fault detection and signal restoration for sensors detecting vehicle motion (e.g., speed, steering angle, and rotational angular velocity sensors), assuming that only one sensor can fail at a given instant, such that the sensor fault detection and restoration algorithm can be applied. In this regard, real-time fault diagnosis and signal estimation of major sensors have been extensively researched [37,38,39,40,41,42,43,44,45,46]; major methods and research trends for fault detection have been introduced and investigated by Miljkovic [47]. When attempting to diagnose the case where several sensors are mounted on a vehicle, residuals are generated for the diagnosis from each sensor and threshold is applied to each diagnosis; here, the number of thresholds is equal to the number of sensors to be diagnosed [18,24,26]. Therefore, each of the corresponding threshold values must be carefully set for the appropriate fault diagnosis of each sensor; otherwise, the result may affect the fault identification of other sensors. In the proposed method, it is possible to identify six sensor faults with only two threshold values and conditional expressions. As a result, the possibility of diagnostic error due to the threshold setting can be considerably reduced.

To prepare for unexpected sensor failure, this study develops a method for signal duplication using the analytical redundancy method based on the information provided by sensors installed in the vehicle and a mathematical vehicle model. Moreover, the analytical redundancy method is employed to realize fault detection and signal restoration.

It is difficult to apply existing research results to other vehicles because existing study have a complex logic structure with different types and numbers of sensors applied to the target vehicle. In addition, existing studies have difficulty in practical application because they have a multi-variable structure in which the judgment logic of failure is complex. These results do not demonstrate the effect of the actual running on the failure of each sensor. Furthermore, their research results do not demonstrate the effectiveness of their performance by applying a restored signal for each fault.

This study aims to detect main sensor faults in real time to guarantee occupants’ safety in a vehicle automatically following a designated route without a driver. A vehicle model-based fault detection and signal restoration method is proposed, and the information provided by the failed sensor is restored to create a time margin in which the vehicle control system can conduct normal safety control procedures. To judge the fault of each vehicle sensor, one first needs to identify a suitable comparison standard. Therefore, the velocity and direction components in the central vehicle axis are defined as the central information on vehicle motion. In the absence of abnormalities in each sensor, the central axis speeds estimated by each vehicle sensor should have the same value. Moreover, if the center speed is correct, it can be used to estimate individual wheel speeds, and the calculated values should match actual wheel speeds if each sensor is normal. A kinematic model is designed to convert the speed of each wheel into the central axis speed. In this process, relationships for estimating the central axis speed using a steering angle or a gyroscope are defined, and fault classification conditions obtained by applying these are derived.

To validate the proposed method, defects are simulated independently for each normal sensor signal. The signal of the normal sensor is compared with the restored signal, and the resulting error is presented. Additionally, it is confirmed that when the fault signal is restored and applied to the position estimation of the autonomous vehicle, the results of path tracking error are within the valid range.

In summary, the contribution of this study is as follows:

- By conducting studies on fault diagnosis and restoration based on the major motion sensors that are installed in most vehicles, the possibility that this study can be used in several vehicles has increased.
- A vehicle kinematic model based on the central axis of the vehicle, which is used to detect faults and restore fault signals using a structure in which the failure of each sensor does not affect each other, is proposed.
- In the logic for fault detection, finally, a simple diagnostic logic structure is presented; this structure helps discriminate between normal and fault and distinguishes a specific fault using a conditional expression of only two variables.

## 2. Fault Detection and Recovery

### 2.1. Configuration of the Autonomous Vehicle

**Figure 1:**
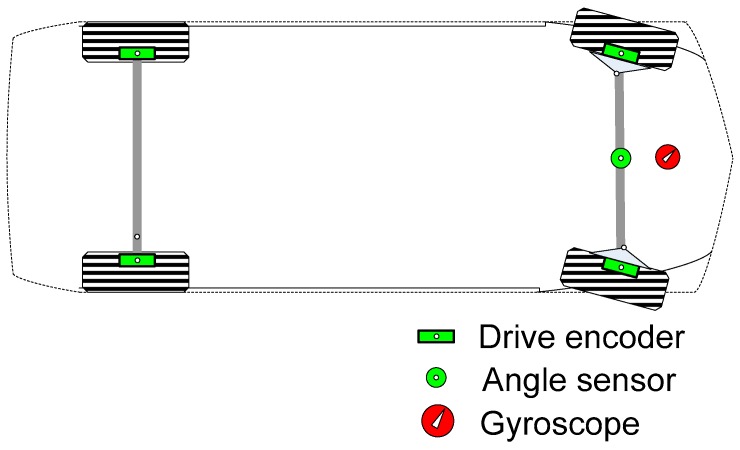
Vehicle sensor configuration.

The target vehicle has four speed sensors, one steering angle sensor, and one gyroscope for vehicle control (Figure 1) [48]. It is assumed that two or more sensors are not defective at the same time, i.e., only one fault is assumed to occur at any moment.

### 2.2. Influence of Sensor Fault

The sensor mounted on the vehicle strongly influences the safety of automatic operation. First, the vehicle controller determines the vehicle position during driving in automatic mode and controls the speed and steering angle to reach the destination based on the fusion of various sensors. The positional information of the vehicle is important for driving control and can be estimated by fusing landmark information, GPS information, and vehicle motion sensor information. The vehicle control system selects a certain point in the vehicle and controls the speed and position of this point to match the desired reference. For example, when the central axis of rear wheels is used as the control reference point in a straight-running vehicle, the calculated speed becomes half of the actual speed if the simple average of the left and right rear wheel speeds is used and one of the corresponding sensors fails. Moreover, when this information is used for position estimation, the calculated position deviates from the actual position. If the gyroscope fails in a curved path, and the information provided by this device is used for position estimation, the vehicle running direction is not calculated correctly, and the resulting position error causes a deviation in route guidance control. Finally, depending on the failure situation, a fault of the steering angle sensor may cause a path-following error or deviation from the traveling path.

### 2.3. Architecture of Fault Detection

**Figure 2:**
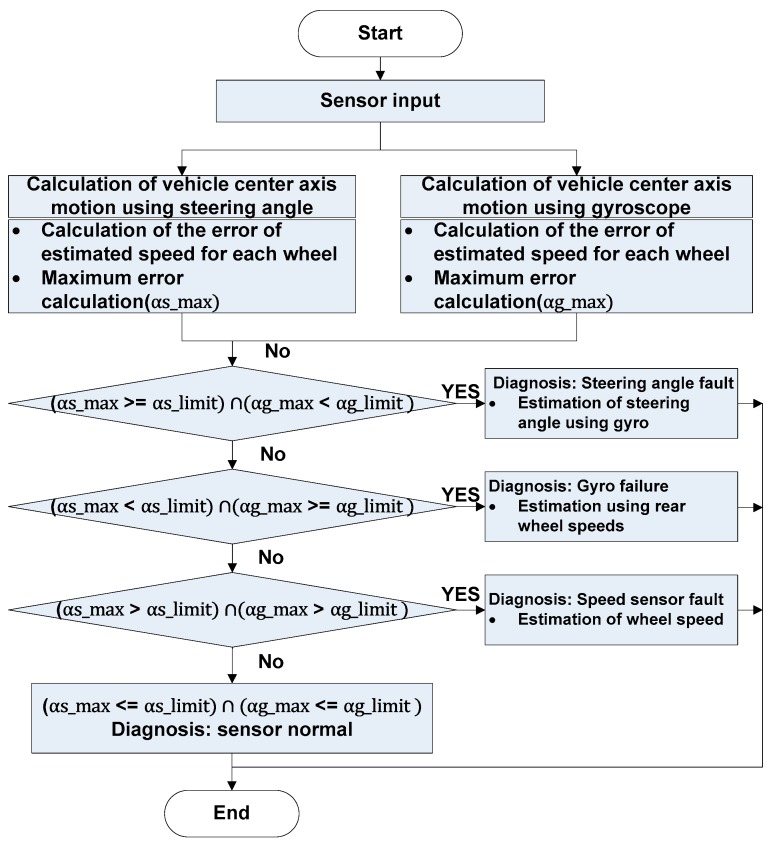
Diagnostic sequence flowchart.

The main idea behind sensor fault detection and signal restoration process is the conversion of the speed of each wheel to the central axis speed of the vehicle. If all sensors are normal, all calculated central axis speeds should be identical, whereas different values should be obtained in the case when any sensor fails. Thus, this difference can be used as a sensor fault indicator. Moreover, once a fault is found, the speed of each wheel can be estimated from the center axis speed determined as normal, and the faulty signal is recovered by replacing the speed of the defective wheel with the estimated speed. In this case, steering angle information is used to convert the speed of each wheel to the central axis speed. If there is an error in the steering angle sensor, the calculated value is also erroneous, which highlights the importance of knowing whether the steering angle sensor is normal or not. Therefore, two methods are used to calculate the central axis speed. Specifically, the input variables are divided into the cases of steering angle usage and usage of gyroscope-provided information on rotational angular speed. This strategy allows one to detect speed sensor, steering angle sensor, and gyroscope faults and to restore the affected signals even if either the steering angle sensor or the gyro sensor is abnormal. The employed procedure is illustrated in Figure 2.

### 2.4. Vehicle Geometry for Kinematic Estimation

**Figure 3:**
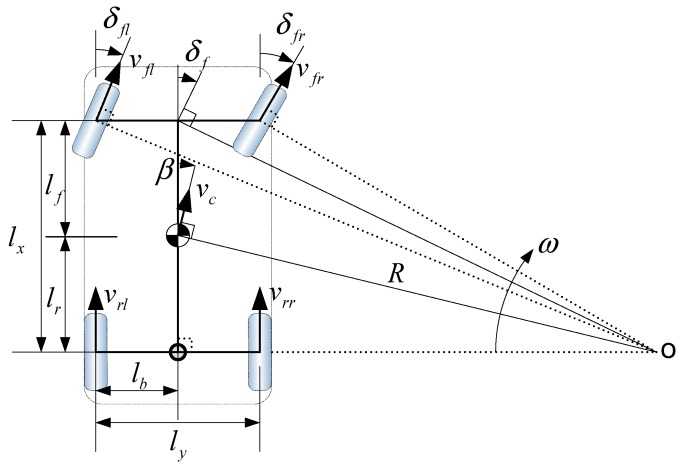
Vehicle geometry for kinematic estimation.

Figure 3 depicts the situation in which the central axis of the vehicle moves around the center of rotation (o) to define the kinematic motion of the vehicle and associated parameters.

In the above figure, *β* is the side slip angle, *δ_f_* is the front wheel steering angle, *δ_fl_* is the front left wheel steering angle, *δ_fr_* is the front right wheel steering angle, *l_f_* is the distance from the front axle to the vehicle central axis, *l_r_* is distance from the rear axle to the central axis, *l_x_* is the distance from the front axle to the rear axle, *l_y_* is front or rear axle width, *l_b_* is the half-width of *l_y_*, *ν*_c_ is the speed at the central axis, *ω* is the yaw rate, *R* is the instant radius at central axis, *ν_fl_*, *ν_fr_*, *ν_rl_*, and *ν_rr_* are speeds of the front left, the front right, the rear left, and the rear right wheels, respectively.

### 2.5. Virtual Redundancy of Sensors and Errors

Formulas to convert the vehicle wheel speed to the central axis speed are proposed. The velocity and direction of the central axis are obtained based on the steering angle or gyroscope information.

#### 2.5.1. Central Axis Speed Estimation Based on Steering Angle

When the steering angle information and gyroscope information are fused together in the fault detection formula, it becomes difficult to distinguish between each of the corresponding faults. Therefore, to differentiate the steering angle fault from the gyroscope fault, the vehicle central axis speed is calculated from the wheel speed, the steering angle, and vehicle parameters excluding the gyroscope information. First, the side slip angle and curvature are obtained from Equations (1) and (2), respectively, using the front wheel steering angle and vehicle parameters.

(1) $$\beta =\tan^{-1}\left(\frac{l_{r}\tan(\delta_{f})}{l_{f}+l_{r}}\right),$$

(2) $$C=\frac{1}{R}=\frac{\cos(\beta )\tan(\delta_{f})}{l_{f}+l_{r}}.$$

**Figure 4:**
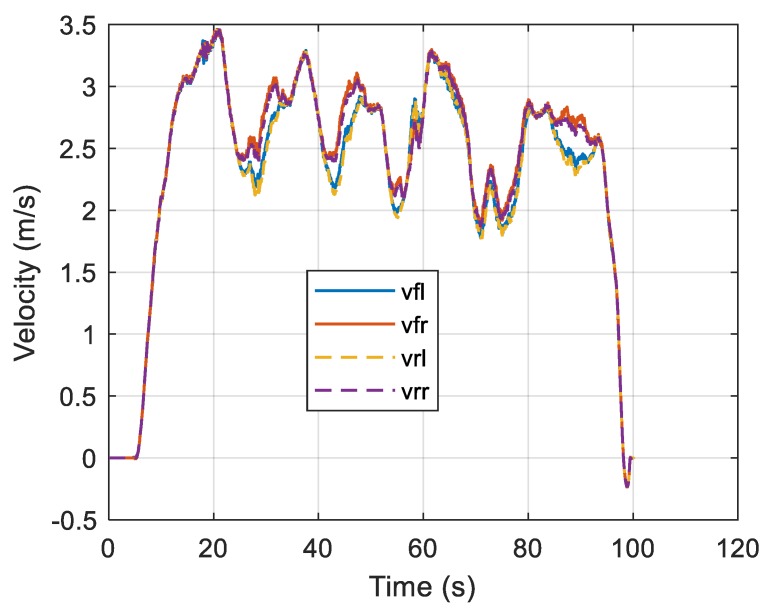
Speeds of the four wheels as functions of time.

The central axis speed can be obtained from Equation (3) to Equation (6) using individual wheel speeds and Equations (1) and (2).

(3) $$v_{c,fl}=\frac{v_{fl}}{\sqrt{{\left(\frac{\cos(\beta )}{\cos(\delta_{f})}\right)}^{2}+l_{b}{}^{2}C^{2}-2l_{b}C\cos(\beta )}},$$

(4) $$v_{c,fr}=\frac{v_{fr}}{\sqrt{{\left(\frac{\cos(\beta )}{\cos(\delta_{f})}\right)}^{2}+l_{b}{}^{2}C^{2}+2l_{b}C\cos(\beta )}},$$

(5) $$v_{c,rl}=\frac{v_{rl}}{\sqrt{{\left(\cos(\beta )\right)}^{2}+l_{b}{}^{2}C^{2}-2l_{b}C\cos(\beta )}},$$

(6) $$v_{c,rr}=\frac{v_{rr}}{\sqrt{{\left(\cos(\beta )\right)}^{2}+l_{b}{}^{2}C^{2}+2l_{b}C\cos(\beta )}},$$

where *C* is the curvature at the central axis, and *ν_c,ij_* is the central axis speed calculated based on individual vehicle wheel data. In a straight section, the speed of each wheel is the same, whereas in a curved section, the speed inside the curvature radius is lower than that on the outside, as shown in Figure 4. 

**Figure 5:**
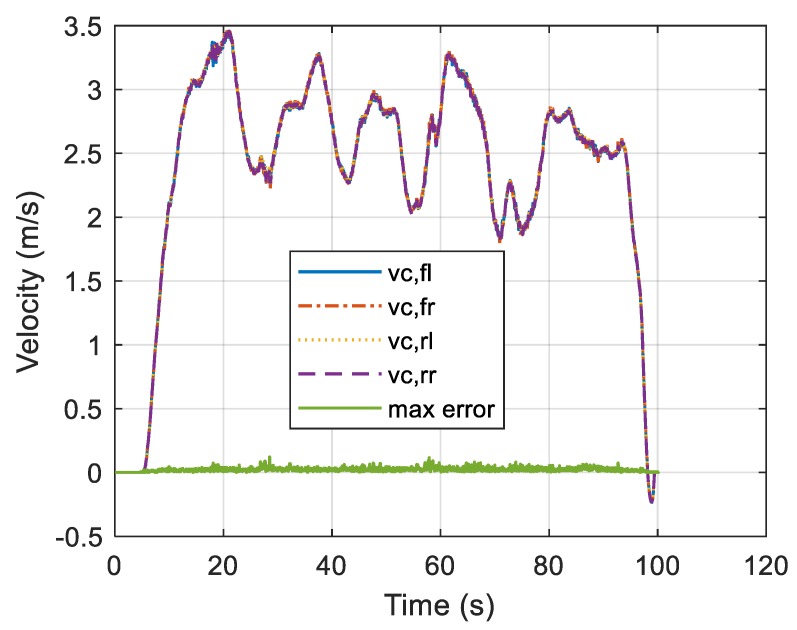
Central axis speeds as functions of time.

The central axis speed satisfies the condition of Equation (7) when all wheel speed and steering angle sensors are normal and hence afford almost identical speed values, as shown in Figure 5. In practice, small differences may occur depending on road conditions and vehicle characteristics, as indicated by the maximum error in Figure 5. Here, maximum error refers to the maximum value of Equation (9). In the vehicle test under normal conditions, the maximum error was measured to be within 0.1 m/s.

(7) $$v_{c,fl}=v_{c,fr}=v_{c,rl}=v_{c,rr}.$$

Therefore, if the central axis speed error exceeds a predetermined threshold value, the sensor or another part are considered to be abnormal, i.e., such an error may be indicative of an abnormal pressure difference between wheel tires, wheel slippage, wheel encoder abnormality, steering angle error, or communication abnormality.

The steering angle information is included in Equation (1) to Equation (6), i.e., if the steering angle information is defective, all calculated central axis speeds may be erroneous. Therefore, if the steering angle information is assumed to be normal and there is a defect in the speed sensor information, the following procedure is followed to select two judged-to-be-normal speeds from the four calculated central axis speeds. The two most closely matched central axis speeds are selected using Equations (8) and (9).

(8) $$(v_{ci,\min},v_{cj,\min})=\min\left(E_{v}\right),\;i\in \left\{fl,\;fr,rl\right\},\;j\in \left\{fr,\;rl,rr\right\},\;i\neq j,$$

with

(9) $$\begin{array}{c} E_{v}=\left[e_{v12}\;e_{v13}\;e_{v14}\;e_{v23}\;e_{v24}\;e_{v34}\right],\\ e_{v12}=\left|v_{c,fl}-v_{c,fr}\right|,\;e_{v13}=\left|v_{c,fl}-v_{c,rl}\right|,\;e_{v14}=\left|v_{c,fl}-v_{c,rr}\right|,\\ e_{v23}=\left|v_{c,fr}-v_{c,rl}\right|,\;e_{v24}=\left|v_{c,fr}-v_{c,rr}\right|,\;e_{v34}=\left|v_{c,rl}-v_{c,rr}\right|. \end{array}$$

The selected two central axis speeds are averaged (Equation (10)) as

(10) $$v_{cr}=\left(v_{ci,\min}+v_{cj,\min}\right)/2.$$

Next, the obtained average is used to estimate the speed of each wheel (Equation (11) to Equation (14)):

(11) $${\hat{v}}_{fls}=v_{cr}\sqrt{{\left(\frac{\cos(\beta )}{\cos(\delta_{f})}\right)}^{2}+l_{b}{}^{2}C^{2}-2l_{b}C\cos(\beta )},$$

(12) $${\hat{v}}_{frs}=v_{cr}\sqrt{{\left(\frac{\cos(\beta )}{\cos(\delta_{f})}\right)}^{2}+l_{b}{}^{2}C^{2}+2l_{b}C\cos(\beta )},$$

(13) $${\hat{v}}_{rls}=v_{cr}\sqrt{{\left(\cos(\beta )\right)}^{2}+l_{b}{}^{2}C^{2}-2l_{b}C\cos(\beta )},$$

(14) $${\hat{v}}_{rrs}=v_{cr}\sqrt{{\left(\cos(\beta )\right)}^{2}+l_{b}{}^{2}C^{2}+2l_{b}C\cos(\beta )}.$$

The thus obtained estimated speed of each wheel should match the measured speed of the wheel if there is no fault in each sensor. For the purpose of fault detection, the error between the estimated and the measured wheel speed is calculated as in Equation (16), and the largest among these errors (defined as in Equation (15)) is used as a condition variable value for detecting sensor defects.

(15) $$as_{\max}=\max\left(E_{vs}\right),$$

with

(16) $$\begin{array}{c} E_{vs}=\left[\begin{array}{cccc} e_{vfl} & e_{vfr} & e_{vrl} & e_{vrr} \end{array}\right],\\ e_{vfl}=\left|{\hat{v}}_{fls}-v_{fl}\right|\\ e_{vfr}=\left|{\hat{v}}_{frs}-v_{fr}\right|\\ e_{vrl}=\left|{\hat{v}}_{rls}-v_{rl}\right|\\ e_{vrr}=\left|{\hat{v}}_{rrs}-v_{rr}\right| \end{array}$$

#### 2.5.2. Central Axis Speed Estimation Using the Gyroscope

To distinguish between steering angle and gyro faults, gyro information is used instead of steering angle information in the calculation of the central axis speed. For this purpose, the steering angle is estimated from the gyroscope information, wheel speed, and vehicle parameters, and the central steering angle is estimated using Equation (17) to Equation (20).

(17) $${\hat{\delta }}_{f1}=\cot^{-1}\left(\cot({\hat{\delta }}_{fl1})+\frac{l_{b}}{l_{x}}\right),\;with\;{\hat{\delta }}_{fl1}=\sin^{-1}\left(\frac{\omega l_{x}}{v_{fl}}\right),$$

(18) $${\hat{\delta }}_{f2}=\cot^{-1}\left(\cot({\hat{\delta }}_{fr1})-\frac{l_{b}}{l_{x}}\right),\;with\;{\hat{\delta }}_{fr1}=\sin^{-1}\left(\frac{\omega l_{x}}{v_{fr}}\right),$$

(19) $${\hat{\delta }}_{f3}=\cot^{-1}\left(\cot({\hat{\delta }}_{fl2})+\frac{l_{b}}{l_{x}}\right),\;with\;{\hat{\delta }}_{fl2}=\tan^{-1}\left(\frac{\omega l_{x}}{v_{rl}}\right),$$

(20) $${\hat{\delta }}_{f4}=\cot^{-1}\left(\cot({\hat{\delta }}_{fr2})-\frac{l_{b}}{l_{x}}\right),\;with\;{\hat{\delta }}_{fr2}=\tan^{-1}\left(\frac{\omega l_{x}}{v_{rr}}\right),$$

where *ω* is the rotational angular velocity measured by the gyroscope. The estimated front-center steering angles should be equal to each other as shown in Equation (21) if there are no faults in the speed sensor and gyroscope.

(21) $${\hat{\delta }}_{f1}={\hat{\delta }}_{f2}={\hat{\delta }}_{f3}={\hat{\delta }}_{f4}=\delta_{f}.$$

If the gyroscope is fault-free, a fault of the speed sensor should result in a difference between some estimated steering values. Therefore, to select the steering angle with the smallest error due to the defect, two estimated steering angles with the smallest error among the estimated steering angles (${\hat{\delta }}_{f1},\;{\hat{\delta }}_{f2},\;{\hat{\delta }}_{f3},\;{\hat{\delta }}_{f4}$) are determined using Equations (22) and (23).

(22) $$\left({\hat{\delta }}_{fi,\min},{\hat{\delta }}_{fj,\min}\right)=\min\left(E_{\delta k}\right),\;i\in \left\{1,\;2,\;3\right\},\;j\in \left\{2,\;3,4\right\},\;i\neq j,\;i\langle j,$$

with

(23) $$\begin{array}{c} E_{\delta k}=\left[e_{\delta 12}\;e_{\delta 13}\;e_{\delta 14}\;e_{\delta 23}\;e_{\delta 24}\;e_{\delta 34}\right],\\ e_{\delta 12}=\left|{\hat{\delta }}_{f1}-{\hat{\delta }}_{f2}\right|,\;e_{\delta 13}=\left|{\hat{\delta }}_{f1}-{\hat{\delta }}_{f3}\right|,\;e_{\delta 14}=\left|{\hat{\delta }}_{f1}-{\hat{\delta }}_{f4}\right|\\ e_{\delta 23}=\left|{\hat{\delta }}_{f2}-{\hat{\delta }}_{f3}\right|,\;e_{\delta 24}=\left|{\hat{\delta }}_{f2}-{\hat{\delta }}_{f4}\right|,\;e_{\delta 34}=\left|{\hat{\delta }}_{f3}-{\hat{\delta }}_{f4}\right| \end{array}$$

The selected estimated steering angles are averaged (Equation (24)), and the obtained value is used as the estimated front wheel steering angle.

(24) $${\hat{\delta }}_{f}=\left({\hat{\delta }}_{fi,\min}+{\hat{\delta }}_{fj,\min}\right)/2.$$

If the front steering angle is estimated using the gyroscope information, a process identical to that used for central axis speed estimation, as described in the previous section, is applied. The speed at each vehicle center is calculated using the speed of each wheel and the estimated steering angle. Next, the side slip angle and curvature are obtained as in Equations (25) and (26), respectively, using the estimated steering angle and vehicle parameters.

(25) $$\beta =\tan^{-1}\left(\frac{l_{r}\tan({\hat{\delta }}_{f})}{l_{f}+l_{r}}\right),$$

(26) $$C=\frac{1}{R}=\frac{\cos(\beta )\tan({\hat{\delta }}_{f})}{l_{f}+l_{r}}.$$

The central axis speed can be obtained using Equation (27) to Equation (30) by considering individual wheel speeds, Equations (25) and (26).

(27) $$v_{c,fl}=\frac{v_{fl}}{\sqrt{{\left(\frac{\cos(\beta )}{\cos({\hat{\delta }}_{f})}\right)}^{2}+l_{b}{}^{2}C^{2}-2l_{b}C\cos(\beta )}},$$

(28) $$v_{c,fr}=\frac{v_{fr}}{\sqrt{{\left(\frac{\cos(\beta )}{\cos({\hat{\delta }}_{f})}\right)}^{2}+l_{b}{}^{2}C^{2}+2l_{b}C\cos(\beta )}},$$

(29) $$v_{c,rl}=\frac{v_{rl}}{\sqrt{{\left(\cos(\beta )\right)}^{2}+l_{b}{}^{2}C^{2}-2l_{b}C\cos(\beta )}},$$

(30) $$v_{c,rr}=\frac{v_{rr}}{\sqrt{{\left(\cos(\beta )\right)}^{2}+l_{b}{}^{2}C^{2}+2l_{b}C\cos(\beta )}}.$$

**Figure 6:**
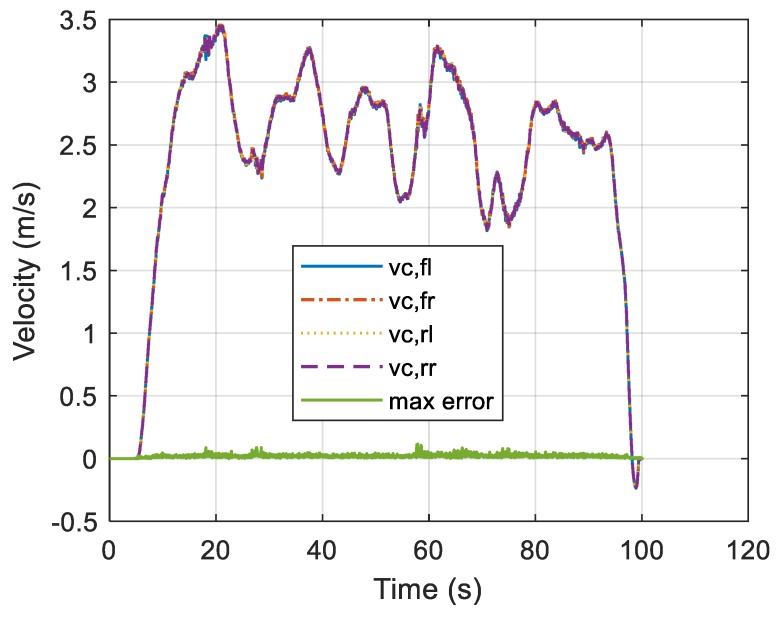
Time-dependent central axis speeds.

If each wheel speed and the gyroscope are normal, the calculated central axis speeds should be equal to each other as shown in Equation (31) (Figure 6).

(31) $$v_{c,fl}=v_{c,fr}=v_{c,rl}=v_{c,rr}.$$

As in the previous case, two speeds with the smallest error selected based on the error between the central axis speed of the two combinations are averaged (Equation (32)).

(32) $$v_{cg}=\left(v_{ci,\min}+v_{cj,\min}\right)/2.$$

The thus obtained value is used to estimate the speed of each wheel using Equation (33) to Equation (36).

(33) $${\hat{v}}_{flg}=v_{cg}\sqrt{{\left(\frac{\cos(\beta )}{\cos({\hat{\delta }}_{f})}\right)}^{2}+l_{b}{}^{2}C^{2}-2l_{b}C\cos(\beta )},$$

(34) $${\hat{v}}_{frg}=v_{cg}\sqrt{{\left(\frac{\cos(\beta )}{\cos({\hat{\delta }}_{f})}\right)}^{2}+l_{b}{}^{2}C^{2}+2l_{b}C\cos(\beta )},$$

(35) $${\hat{v}}_{rlg}=v_{cg}\sqrt{{\left(\cos(\beta )\right)}^{2}+l_{b}{}^{2}C^{2}-2l_{b}C\cos(\beta )},$$

(36) $${\hat{v}}_{rrg}=v_{cg}\sqrt{{\left(\cos(\beta )\right)}^{2}+l_{b}{}^{2}C^{2}+2l_{b}C\cos(\beta )}.$$

The thus estimated speeds of each wheel should match the measured values if there is no defect in each sensor. For the purpose of fault detection, the error between each estimated wheel speed and the measured wheel speed is obtained as in Equation (38), and the largest among these errors (defined as in Equation (37)) is used as a condition variable value for detecting sensor faults.

(37) $$ag_{\max}=\max\left(E_{vg}\right),$$

with

(38) $$\begin{array}{c} E_{vg}=\left[\begin{array}{cccc} e_{vfl} & e_{vfr} & e_{vrl} & e_{vrr} \end{array}\right],\\ e_{vfl}=\left|{\hat{v}}_{flg}-v_{fl}\right|\\ e_{vfr}=\left|{\hat{v}}_{frg}-v_{fr}\right|\\ e_{vrl}=\left|{\hat{v}}_{rlg}-v_{rl}\right|\\ e_{vrr}=|{\hat{v}}_{rrg}-v_{rr}|. \end{array}$$

### 2.6. Fault Detection, Identification, and Signal Recovery

To identify sensor faults, Equations (15) and (37), which describe the calculation of maximum estimated speed and the measured speed error, are used for judgment. The maximum values of each error (*as*_max_, *ag*_max_) and the corresponding limit values (*as*_limit_, *ag*_limit_) are used to determine whether the sensor is faulty.

#### 2.6.1. Fault-Free Sensor Judgment Condition

If the maximum error (*as*_max_, *ag*_max_) between the estimated speed and the measured speed is small, all sensors applied to the relational expression can be viewed as not defective. The maximum allowable error limit (*as*_limit_, *ag*_limit_), which is the defect judgment boundary, is determined based on the observed results under the condition that all sensors are normal. Herein, limit values of *as*_limit_ = *ag*_limit_ = 0.025 were selected for the running test of the test vehicle. Therefore, if the maximum error (*as*_max_, *ag*_max_) is smaller than the above values, all sensors are normal, and if not, a fault is concluded to be present. This condition is expressed in Equation (39).

(39) $$\begin{array}{l} if\;(as_{\max}\leq as_{\mathrm{limit}})\cap (ag_{\max}\leq ag_{\mathrm{limit}})\\ \;TRUE:Normal\\ else\\ \;FALSE:Other\;fault\\ end \end{array}$$

#### 2.6.2. Fault Detection and Signal Restoration for Steering Angle Information

If the maximum value (*ag*_max_) of the error calculated using gyroscope information is smaller than the limit value (*ag*_limit_), the gyroscope and speed sensor are both viewed as normal. If the maximum value (*as*_max_) of the error calculated using steering angle information is larger than the limit value (*as*_limit_), the steering angle sensor is viewed as defective, as there is no defect of the speed sensor under the preceding condition. This condition is expressed in Equation (40).

(40) $$\begin{array}{l} if\;(as_{\max}\geq as_{\mathrm{limit}})\cap (ag_{\max}\langle ag_{\mathrm{limit}})\\ \;TRUE:Steering\;sensor\;fault\\ end \end{array}$$

If a steering angle fault is identified according to the condition of Equation (40), the measured steering angle sensor information is replaced with the estimated steering angle information calculated using Equation (24).

#### 2.6.3. Gyroscope Fault Detection and Signal Restoration

If the maximum value (*as*_max_) of the error calculated using steering angle information is smaller than the limit value (*as*_limit_), both the steering angle sensor and the speed sensor are viewed as normal. In this case, if the maximum value (*ag*_max_) of the error calculated using the gyroscope information is larger than the limit value (*ag*_limit_), one can judge that only the gyroscope is defective, as it follows from the above that the speed sensor is not defective.

(41) $$\begin{array}{l} if(as_{\max}\langle as_{\mathrm{limit}})\cap (ag_{\max}\;\geq \;ag_{\mathrm{limit}})\\ \;TRUE:Gyroscope\;fault\\ end \end{array}$$

If a gyroscope fault is identified according to the condition of Equation (41), rotational angular velocity is estimated from Equation (42) by applying Equations (13) and (14), which describe the estimation of rear wheel speeds based on steering angle information.

(42) $$\hat{\omega }=\frac{{\hat{v}}_{rrs}-{\hat{v}}_{rls}}{2l_{b}}.$$

#### 2.6.4. Fault Detection of the Speed Sensor and Signal Restoration 

Under the condition that the gyroscope and the steering angle sensor do not fail simultaneously, the speed sensor is viewed as defective if (i) the maximum value (*as*_max_) of the error calculated based on steering angle information is greater than the limit value (*as*_limit_), and (ii) the maximum value (*ag*_max_) of the error calculated based on the gyroscope information is greater than the limit value (*ag*_limit_) [the error of the speed sensor affects both sides]. In this case, when the speed error is larger than the limit value, fault identification of each speed sensor can be performed as follows.

(43) $$\begin{array}{l} if(as_{\max}\;\rangle \;as_{\mathrm{limit}})\cap (ag_{\max}\;\rangle \;ag_{\mathrm{limit}})\\ \;TRUE:Speed\;encoder\;fault\\ \;if\;(e_{vfl}\rangle as_{\mathrm{limit}})\\ \;TRUE:Front\;left\;speed\;encoder\;fault\\ \;end\\ \;if\;(e_{vfr}\rangle as_{\mathrm{limit}})\\ \;TRUE:Front\;right\;speed\;encoder\;fault\\ \;end\\ \;if\;(e_{vrl}\rangle as_{\mathrm{limit}})\\ \;TRUE:Rear\;left\;speed\;encoder\;fault\\ \;end\\ \;if\;(e_{vrr}\rangle as_{\mathrm{limit}})\\ \;TRUE:Rear\;right\;speed\;encoder\;fault\\ \;end\\ end \end{array}$$

If a speed sensor fault is identified according to the condition of Equation (43), the value provided by the defective sensor is replaced with the value estimated using Equation (11) to Equation (14).

## 3. Results and Discussion 

To verify the validity of the proposed algorithm, we performed an off-line test using the sensor data collected from the test vehicle [48]. The vehicle was set up to run in loop guided 235-m test tracks in automatic path guided mode, and sensor information was collected during driving. The vehicle control system collected the signal of magnetic markers embedded in the road and combined it with vehicle motion sensor information to determine real-time vehicle location/orientation and perform automatic guidance control. Faults, failures, and malfunctions observed during driving (e.g., those of the speed sensor, the steering angle sensor, and the rotational angular velocity sensor) may result in erroneous normal position estimation, guidance control errors, or deviation from the suggested path. The results of the off-line test showed how each sensor fault affects vehicle control. In addition, the validity of the proposed method was verified by identifying the defective sensor and replacing the corresponding signal with the estimated value to show whether one can maintain the normal traveling orbit within the effective error range.

### 3.1. Speed Sensor Fault Test

**Figure 7:**
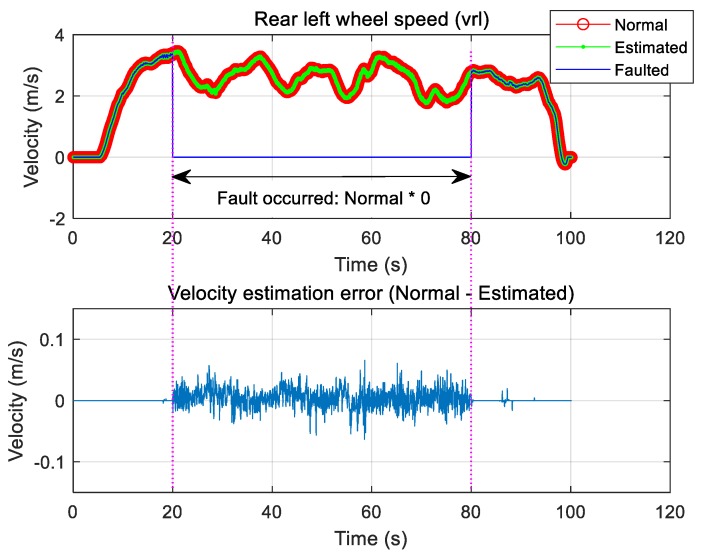
Type 1: Rear left wheel speed sensor fault and estimation.

**Figure 8:**
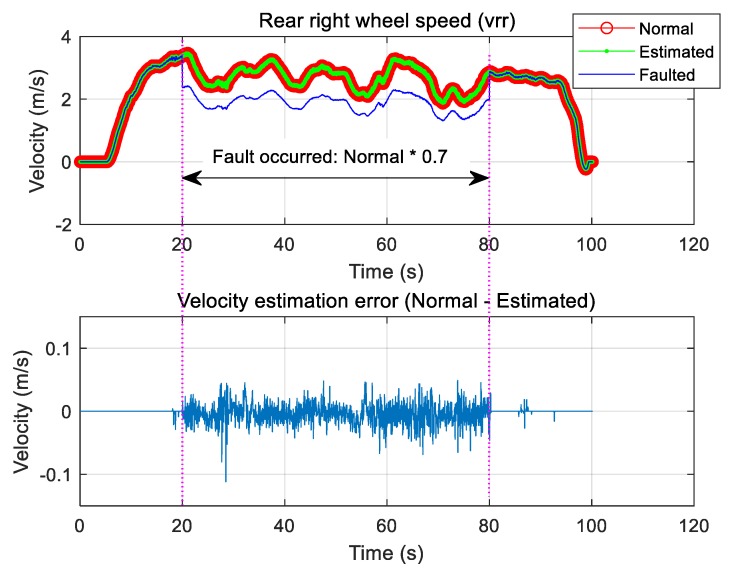
Type 2: Rear right wheel speed sensor fault and estimation.

One of the four wheels simulated the fault of one rear right wheel speed sensor from 20 to 80 s. A zero was injected during this time instead of the normal signal to simulate a failure. Therefore, the actual speed was known. The faulted signal, the normal signal, and the signal estimated using Equation (13) are shown in Figure 7, which compares the estimated signal and the normal signal to show that the sensor signal can be well estimated even if it changes to zero because of failure simulation, i.e., the normal signal is well restored by the estimator. In this case, the error between the normal value and the estimated value was within 0.05 m/s. For simulated faults, the rear right wheel speed signal decreased to 70% of value of the normal signal during the same time interval. Figure 8 compares the estimated signal with the normal signal for the case in which the measured signal decreased from normal to 70% when tire pressure loss or puncture was assumed.

### 3.2. Steering Angle Sensor Fault

**Figure 9:**
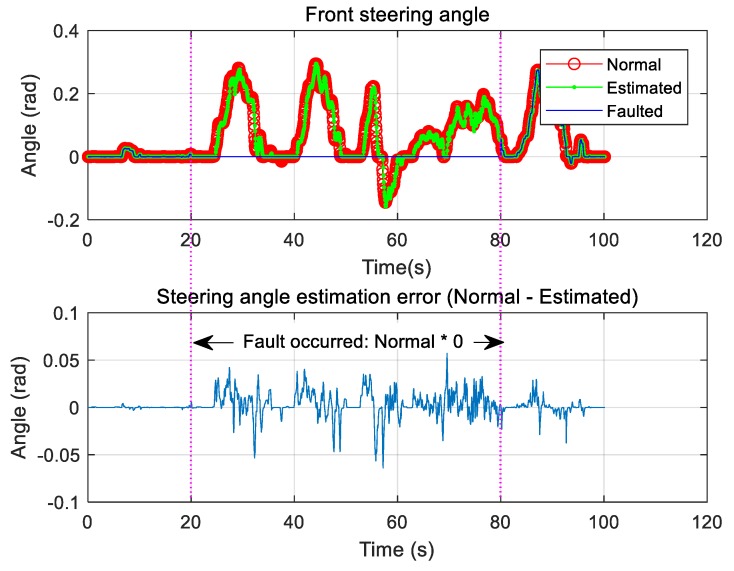
Type 1: Front steering angle sensor fault and estimation.

**Figure 10:**
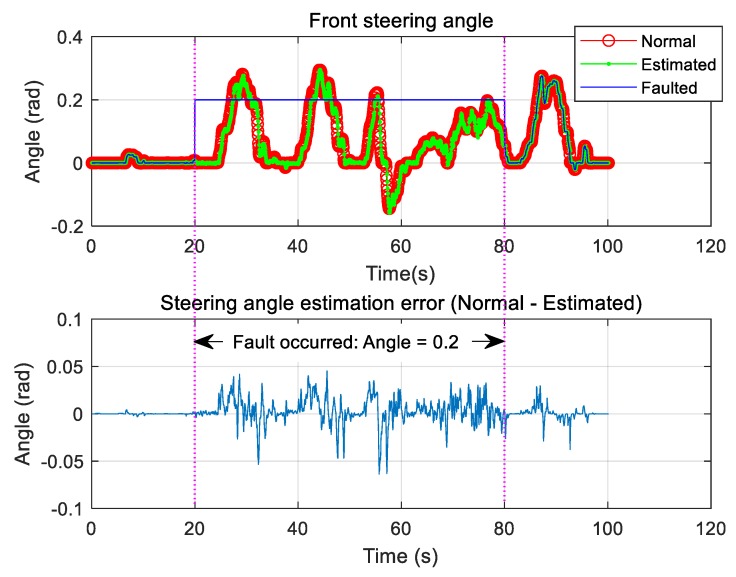
Type 2: Front steering angle sensor fault and estimation.

Only the case where the steering sensor is faulty was considered, and the fault was chosen to occur between 20 and 80 s. During this period, the steering angle information was changed to zero, and the signal was estimated using the proposed algorithm and compared with the normal sensor signal (Figure 9). Because of the fault, the steering angle sensor value was fixed at a constant value for the same time period. The estimated steering angle information was found to be in good agreement with the normal steering angle information (Figure 10).

### 3.3. Gyroscope Fault

**Figure 11:**
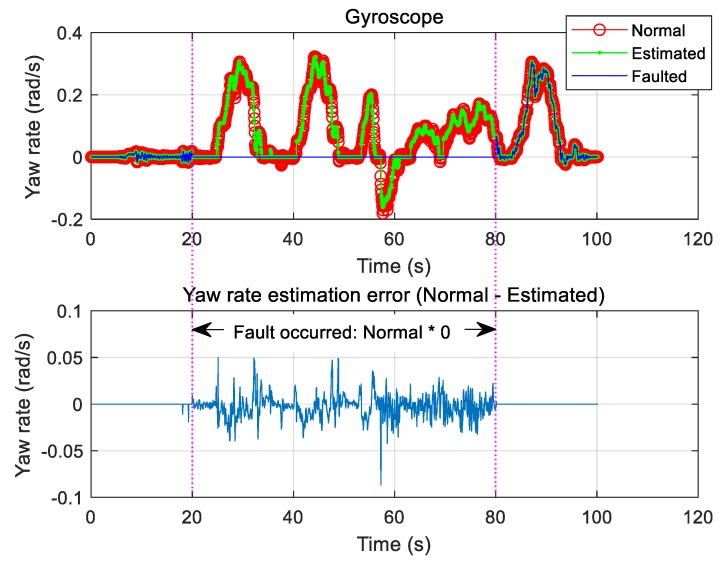
Type 1: Gyroscope fault and estimation.

**Figure 12:**
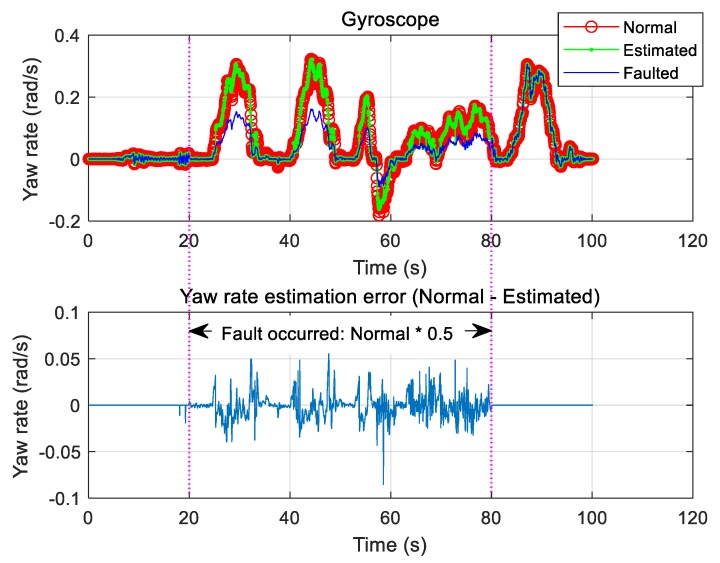
Type 2: Gyroscope fault and estimation.

Only the case where the gyroscope is faulty was considered, and the fault was chosen to occur between 20 and 80 s. During this period, the signal was estimated using the proposed algorithm and compared with the normal sensor signal. Notably, the proposed method allowed for good recovery of the normal signal. Figure 11 shows the result of estimation based on Equation (42). We also tested the scenario of a fault when the signal decreased to 50% of the normal signal over the same time interval. In this case, good signal estimation results were also observed (Figure 12).

### 3.4. Influence of Defects in Automatic Running

This test aimed to check the effect of sensor faults on automatic running and to determine whether the proposed fault detection and signal restoration method is valid. In the case of a running vehicle, the sensor was configured so that only one fault occurs at the same time.

#### 3.4.1. Fault-Free Driving

**Figure 13:**
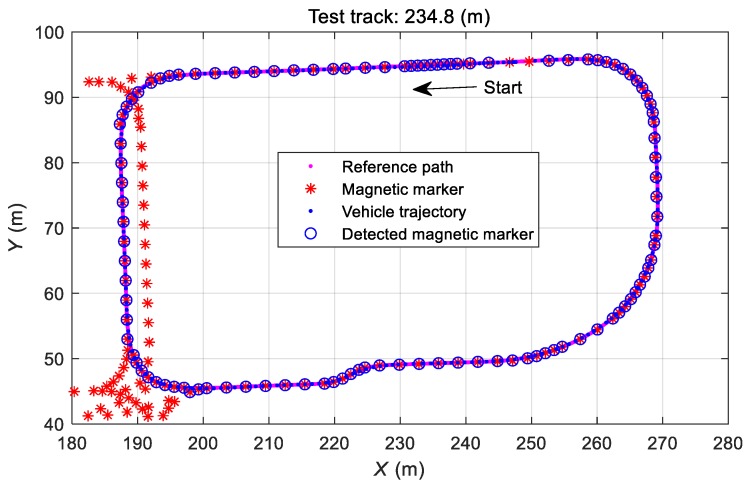
Normal automatic path tracking.

The estimated position and tracking control state of the vehicle were checked in the case where all sensors were normal, and the observed performance was compared to that in the case of sensor fault. Figure 13 shows the driving trajectory of a vehicle that automatically ran the designed path with that of the fault-free vehicle.

#### 3.4.2. Effect of Rear Left-Wheel Speed Sensor Defect during Automatic Driving

**Figure 14:**
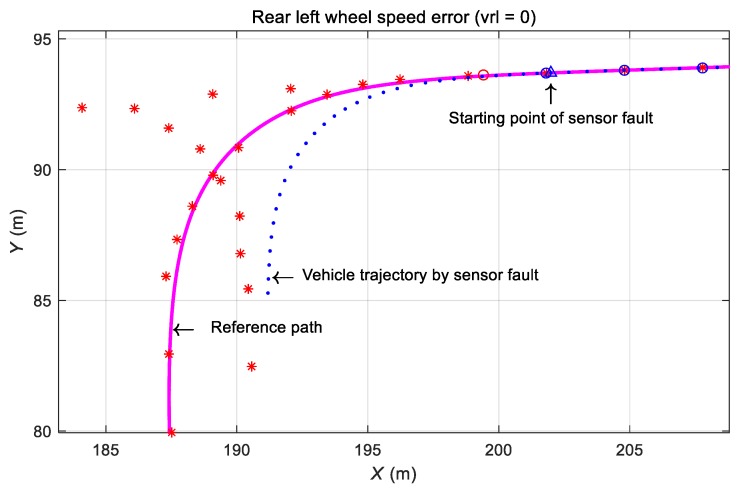
Test result for a wheel speed sensor fault.

The case when only the rear left wheel speed sensor of the vehicle is defective was considered. When the average of the left- and right wheel speed is used as the center speed, the calculated travel distance is half of the actual travel distance when one of the speed sensors fails during vehicle operation, which results in erroneous position calculation and deviation from the traveling path (Figure 14). As shown in Figure 14, when the speed sensor error occurred before the curve was entered, the vehicle trajectory gradually deviated toward the inside of the reference path.

#### 3.4.3. Effect of Gyroscope Defect during Automatic Driving

**Figure 15:**
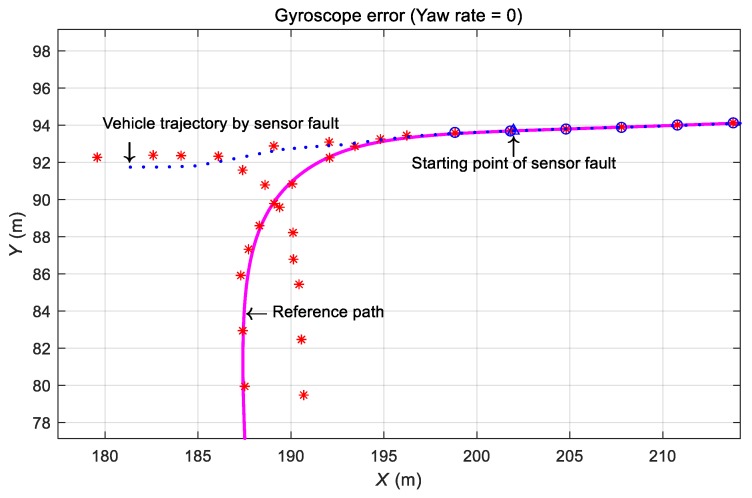
Test result for a gyroscope fault.

When a gyroscope fault occurs during vehicle operation, an error is generated in the calculation of the running direction, which increases the error in the calculation of vehicle position. As a result, the vehicle deviates from the traveling path. As shown in Figure 15, the vehicle was not able to follow the travel route because the state of the rotational angular velocity sensor was “zero” before the curve was entered, and a straight line trajectory was generated.

**Figure 16:**
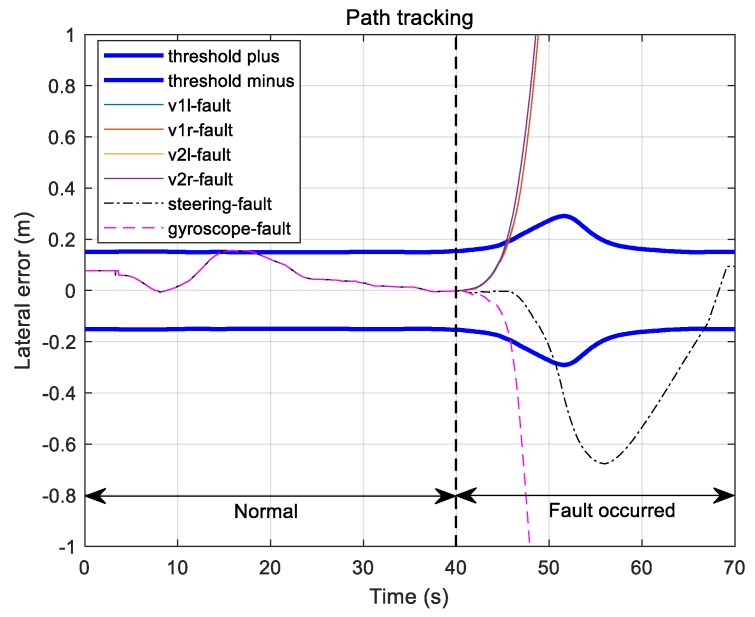
Path tracking errors for each sensor fault.

Figure 16 shows path-following errors of the vehicle under the fault condition of each sensor. The upper and lower thick solid lines in this figure are the maximum allowable travel error boundaries on normal driving, equaling 15 cm on the straight line portion and increasing along the curved portion. Until 40 s before entering the curved section, the vehicle followed the trajectory with an error within 15 cm, but after 40 s, it deviated from the set error margin according to the fault of each sensor.

**Figure 17:**
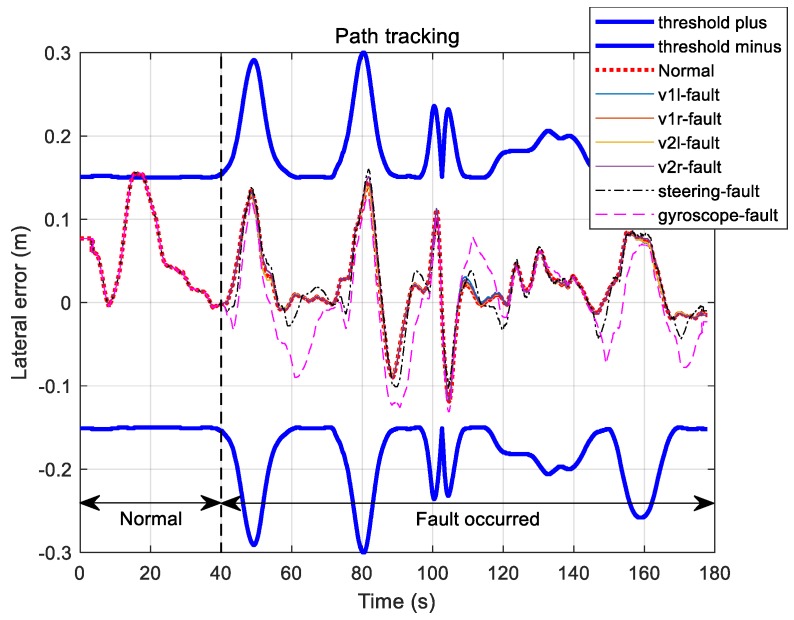
Path tracking errors for each sensor fault recovery.

Figure 17 shows the travel path follow-up error for fault signal restoration under each fault condition. Fault detection and signal restoration were performed 40 s after each sensor fault to afford a running result within the allowable limit error for the normal running of the vehicle. Consequently, the effectiveness of fault detection and the recovery method was successfully verified.

## 4. Conclusions

In this paper, fault diagnosis logic and signal restoration algorithms for vehicle motion sensors are presented. To this end, a central axis kinematic model is applied to each wheel speed of the vehicle. Both steering angle and gyroscope information are considered to distinguish failure effects. For fault diagnosis logic, we derive a conditional expression with only two variables to distinguish between normal and fault, and an analytical redundancy structure and a simple diagnostic logic structure are presented to distinguish specific faults. This study further assumes that only one sensor can fail at any given instant, which may limit the current scope of application of the proposed fault diagnosis scheme.

To verify the validity of this method, vehicle sensor data are collected under normal driving conditions, and the algorithm used in the actual vehicle is applied to estimate the vehicle position and orientation in an off-line test. The risk of automatic driving according to each failure is examined through the addition of faults to normal sensor information. It is shown that the autonomous vehicle can satisfy the valid normal driving conditions when the proposed fault detection and signal restoration method is applied under fault conditions. To improve vehicle safety, the authors plan to investigate diagnostic methods for multiple sensor faults.
